# Learning from Primary Health Care Centers in Nepal: reflective writings on experiential learning of third year Nepalese medical students

**DOI:** 10.1186/s13104-015-1727-2

**Published:** 2015-12-01

**Authors:** Rolina Dhital, Madhusudan Subedi, Neeti Prasai, Karun Shrestha, Milan Malla, Shambhu Upadhyay

**Affiliations:** Department of Community Health Science, Patan Academy of Health Sciences, G P O Box 26500, Kathmandu, Nepal; School of Medicine, Patan Academy of Health Sciences, G P O Box 26500, Kathmandu, Nepal

**Keywords:** Kolb’s experiential learning theory, Medical education, Medical students, Nepal, Primary health care, Reflective writing

## Abstract

**Background:**

Medical education can play important role in cultivating the willingness among the medical students to work in underprivileged areas after their graduation. Experiential learning through early exposure to primary health care centers could help students better understand the opportunities and challenges of such settings. However, the information on the real experiences and reflections of medical students on the rural primary health care settings from low-income countries like Nepal are still limited. The aim of this study is to demonstrate the learning process of the medical students through their reflective writings based on Kolb’s theory of experiential learning.

**Methods:**

The students wrote their experiences, observations and reflections on the experiential learning from the primary health care centers on individual logbook as part of their community posting assignments. We analyzed the data of 50 logbooks through content analysis using Kolb’s experiential learning cycle as a theoretical framework.

**Results:**

The students’ reflections are structured around the four main learning stages of Kolb’s experiential learning theory. Each learning stage consisted of different categories. The first stage consisted of concrete experiences on rural health and learning by doing. The second stage included their reflective observations on primary versus tertiary care, application of theoretical knowledge and role of supervisors. In the third stage, the students developed and refined their concepts on self-development, understanding reality, compassion and sense of responsibility. The final stage, active experimentation, included their immediate future plans, suggestions to improve curriculum, plans after becoming a doctor and suggestions to improve policies.

**Conclusion:**

This study provided important insights on different stages of experiential learning of medical students on primary health care in low resource rural settings. Reflective writing of experiential learning could be an important step to address the gaps in medical education for resource constraint settings like that of Nepal and other low-income countries.

## Background

Globally, medical education focuses more on teaching and training in tertiary care settings where the latest technology, diagnostic tools and expert back up are easily available [1]. On the other hand, lack of sufficient human resources providing quality health care to the poorest and remotest places remain a major global health challenge [2]. Moreover, both the developed and developing parts of the world have been facing the challenges to recruit and retain medical doctors in rural areas [3–6].

The situation is far worse in middle and low-income countries, with 47 % of WHO member states reporting less than one doctor per 1000 population [7]. Nepal is one such low-income country in South Asia facing workforce crisis in healthcare, particularly in the rural areas [8]. In 2012, the number of doctors in Nepal was as low as 0.17 per 1000 population [9]. Over the last decade, Nepal has witnessed a dramatic increase in the production of medical graduates, with more than 1000 medical doctors registering at the Nepal Medical Council every year [10]. Despite the increase in the number of medical graduates, most remain concentrated in the urban areas [5].

In order to familiarize the medical students with the health system in general and rural community health in particular, it is mandatory for all the medical institutions in Nepal to incorporate community medicine or community health sciences in their undergraduate curriculum [11]. Also other south Asian countries like India, Bangladesh, and Pakistan have incorporated community medicine in their undergraduate curriculum [12–14]. However, the way the course is delivered may vary among different institutions and countries. The exposure to the health system and to rural health in particular may not be adequate or uniform among all institutions. Moreover, the information about the students’ real experiences from being exposed to rural health care issues in their medical education, remain scant.

Reflective writing can be an important way to understand the medical students’ learning process in their experiential learning [11, 15]. Written reflections can help the students to think critically, increase their active involvement in learning, and increase their personal ownership of each learning stage [16]. It has been suggested that reflection can help health professionals to understand complex situations and enable them to learn from such experiences [15]. Although gaining popularity in medical education in many parts of the world [17, 18], such practice is still uncommon in most low-income countries. However, it is argued that reflective writing of experiential learning becomes effective only if it is applied appropriately based on a theoretical framework [19].

### Experiential learning theory

In 1984, David Kolb described a four-stage experiential learning cycle, suggesting that a combination of experience and subsequent reflection is important for ‘real’ learning [20]. Reflection is a concept used by many constructivists over the past decades [21–23]. Constructivism is a theory about how people construct their own knowledge and understanding of the world by experiencing and then reflecting upon those experiences [24]. Kolb’s experiential cycle remains the most thoroughly explained and popular theory [25]. It includes four stages: concrete experience, reflective observation, abstract conceptualization, and active experimentation [20, 25].

In Kolb’s first stage, the learners have a concrete experience [20]. This can be an event or just a simple experience, which has the potential to add or change the learner’s knowledge or skills. In the second stage, the learner deliberately tries to review the real experiences in order to understand their value [20]. In the third stage, the learners go through a deeper reflective process, and transform the past experiences into new conceptions or knowledge [20]. In the fourth stage, active experimentation, the students plan to translate the new knowledge into action. This could be either in the form of concrete actions or proposals, which could then lead to a new cycle of learning [20, 25].

**Fig. 1:**
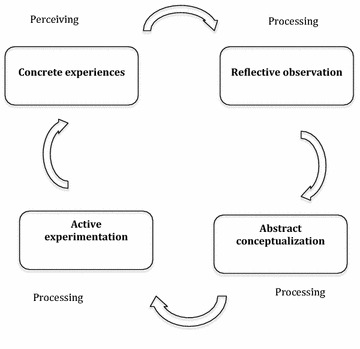
Kolb’s experiential learning cycle

Even though experiential learning has been widely discussed and implemented over the years in medical education, little research has been done to demonstrate each process, particularly in resource constraint settings [25]. Thus, the aim of this study is to demonstrate the stages of experiential learning process of the medical students through their reflective writings based on Kolb’s theory of experiential learning [20] (Fig. 1).

## Methods

### Study setting

Patan Academy of Health Sciences (PAHS), School of Medicine was established in 2010 as an autonomous not-for-profit institution in Nepal, with the purpose of preparing a new generation of medical graduates to work in rural areas. Problem based learning (PBL) and community based learning and education (CBLE), are the principal pedagogic strategies of its medical education program. A significant portion of the CBLE is delivered through mandatory experiential learning in diverse community settings that have different tiers of health facilities within the national health system.

In October 2013, a total of 55 third-year medical students, the first cohort at PAHS, were posted in seven rural Primary Health Care Centers (PHCCs) in two districts for a total duration of 4 weeks. The PHCCs in Nepal is the first level health facility with the provision of at least one medical doctor. The health services provided by the PHCCs where the students were placed included essential primary care like maternal and child health, family planning and outreach services.

In addition to clinical exposure at the PHCCs, the students were required to maintain a written log of their daily work, experiences, and reflections. The students were encouraged to think critically and write their reflections about community health experiences since their first year of medical school. In order to encourage students to write freely, logbooks were not evaluated on the basis of the contents of their writing. All the writings were in English, being the official language for medical education in Nepal.

### Ethical considerations

We obtained ethical approval to use the students’ logbooks for this study from Institutional Review Committee of PAHS. Fifty students provided written informed consent to analyze the written contents from the logbooks.

### Data analysis

Data analysis was done through a qualitative content analysis using Kolb’s categories [26]. To reduce bias and secure anonymity the personal identification of the students such as name, gender, and roll number written on the cover page of each logbook was first covered. Each logbook was then given a code number. The total logbooks were then divided among four researchers (RD, NP, KS and MM). The four researchers then identified any activity, observation, or reflection that the student had mentioned in the logbook. From the logbooks, the hand written information based on these categories was then entered into Microsoft Word. The information entered based on these categories were then verified by two researchers (MS and SU). The principle investigator then further performed content analysis, discussing with all the group members. The broad categories were divided into subcategories that were then fit into themes according to Kolb’s different stages. Anonymous original quotes that reflected the experiences and expectations from the students were chosen to give more insight into the students’ perceptions.

## Results

The students’ reflections are structured around Kolb’s four main learning stages, each stage including different subcategories (Table 1).

**Table 1:** Different stages of experiential learning and categories

Stages	Categories	Sub categories
1. Concrete experiences	Activity, observations, experiences	Experiencing rural health
		Learning by doing
2. Reflective observations	Reflections based on observations	Primary care vs. tertiary care
		Application of theoretical knowledge
	Reflections based on review of experiences	
		Role of supervisors
3. Abstract conceptualization	Reflections from past experiences to new knowledge/concepts	Self development
		Understanding the reality
		Compassion
		Sense of responsibility
4. Active experimentation	Reflections on immediate plans, future plans, suggestions	Immediate future plans
		Suggestions to improve curriculum
		Plans after becoming a doctor
		Suggestions for improving policies

### Stage 1: concrete experiences

#### Experiencing rural health

In this study, almost all the students explained about their observations of the patients in the PHCCs. They mentioned that they were able to witness the challenges the patients had to face.

“I saw a lady presented with post partum hemorrhage following home delivery. We were told that patient was on her way to hospital to deliver the child but some villagers told her to deliver at home. She then returned home to deliver her baby and had to face the complications.” (Code No. 38)

“I saw a case of a young female who presented with history of vaginal bleeding. I found out that she had used over the counter drugs from a local medical store for abortion. She told us that she was a schoolteacher but she did not know about abortion being legal and was afraid if others would know about the termination.” (Code No. 34)

“A mother and her daughter had walked 6 h to reach the health facility. Mother had carried her one year old son all the way. They opted walking because transportation cost is expensive. Instead of paying for the bus she would rather feed her six children.” (Code No. 5)

#### Learning by doing

Almost all the students mentioned about their experiences on learning by doing and stated about the experiences they had in the PHCCs.

“With the help of the nurse, I listened the fetal heart sound through a fetoscope for the first time.” (Code No. 34)

“I learnt to examine a child of under-five years according to IMCI (Integrated Management of Childhood Illness) system.” (Code No. 26)

“I participated in vitamin A and Albendazole distribution program in the community.” (Code No. 36)

### Stage 2: reflective observations

Almost all the students reflected further on their observations and experiences.

#### Primary care versus tertiary care

Many reflected that rural clinical practice in real life differed from the textbooks. They also noted that the patient management was different from what they had observed in the tertiary hospital.

“We have been taught according to standard protocol at the hospital but here at PHCC due to lack of resources it was performed in minimal setting.”(Code No. 9)

“I observed how the staffs working at the PHCC made the best use of available resources for providing the best service possible to the patients.” (Code No. 44)

“In the rotation at the medicine department in the hospital, we were clearly taught how to manage hypertension emergency, but in the community we don’t have all the facilities to manage it as mentioned in the theory.”(Code No. 43)

#### Application of theoretical knowledge

Many reflected that they were able to apply their theoretical knowledge to the real life settings. The students were able to connect the past knowledge to their current experiences.

“I saw a patient with a complaint of vomiting associated with multiple episodes of loose stools with epigastric pain. This case was a good learning opportunity for me as I went back to review clinical practice lessons learned from surgery department in the hospital.” (Code No. 16)

“I have learned to examine pregnant mothers. Though we had studied in theory about pelvic grip, I did not have opportunity to practice so well at our college. I could now perform pelvic grips.” (Code No. 27)

#### Role of supervisors

Almost all the students reflected the importance of faculty to inspire and motivate them to learn in their experiential stay.

“Our faculty visited us and gave us feedback on many things…I felt so good to listen what he was saying that I didn’t want the session to stop. He is such a loving and caring teacher, very empathetic and so into community and really inspires me a lot.” (Code No. 4)

“I was so happy and touched when our teacher understood our feelings. He counseled all of us and made us feel better.” (Code No. 51)

“Our teacher mentioned that we should be creative, innovative writer and for some reason his words have stuck my mind deeply, letting me to write openly what I have in my mind.” (Code No. 45)

However, some students were critical about the mismanagement, lack of timely communication and proper coordination from the faculty.

“We were sad that our teachers did not communicate well with us on issues related to unavailability of vehicles and delays.” (Code No. 5)

“The faculties were supportive but I think what I expected was more.” (Code No. 53)

“I think communication was lagging somewhere. I am sure our faculties will be cautious and prevent this miscommunication in other postings.”

Almost all the students wrote that the local supervisors who were the health workers in the health centers had an important role to play in their learning.

“I realized that for a good learning process, it is important that both local supervisor at PHCC and the students should be enthusiastic about learning.” (Code No. 41)

“I am very impressed and encouraged seeing honesty and dedication of the auxiliary health worker to his work. He is not only a dedicated health worker but also a dedicated teacher to us. From him, I have learnt that I should be honest and dedicated to my work.” (Code No. 17)

“I got to learn so much from the doctor at PHCC. He taught us many things that will be useful for me in the coming examination.” (Code No. 13)

### Stage 3: abstract conceptualization

Almost all the students reflected on how the experiences and reflective observations affected them at a personal level. The students were able to develop or refine their concepts on various aspects by connecting their prior experiences.

#### Self development

Many mentioned that the experiences at PHCC helped them to develop many skills and qualities on their own.

“It has already been 11 days of stay in this PHCC and through this phase, I have gained more confidence in management of patients.” (Code No. 50)

“Today, I could do the urinalysis using my knowledge. Now I have the confidence of doing the urinalysis when I will be posted in rural areas.” (Code No. 35)

Many reflected that the exposures they gained about rural life from the PHCC postings helped them to improve their adaptation skills.

“I was born a city girl and everything is totally different from what we are used to. This posting has certainly made me strong enough to adjust in different settings. These are not just part of the curriculum I am following, these are going to be the milestones towards my self development.” (Code No. 5)

“At a point when it felt like “oh! It’s difficult being here”, unknowingly we started embracing the place and things started getting simpler.” (Code No. 50)

#### Understanding the reality

Many reflected on their helplessness and frustrations by understanding the realities of the resource constraint settings and the challenges people have to face.

“I realized that it is not always possible to practice everything in the community that we have learnt in our hospital. So we should be cautious about resources available and their use.” (Code No. 43)

“It’s inequality. It’s injustice. It’s just a sample, many poor/disadvantaged people are dying in our country because they are unable to get the treatment.” (Code No. 7)

“It is still a tragedy that people travelled for hours to and back home on foot to have themselves checked. This is an unavoidable geographical difficulty and I see no easy solution to this.” (Code No. 50)

“The cancer had already metastasized and had small hole for emphysema of thorax drainage on his left chest. Seeing this patient and his condition, I became emotional and asked myself “what is life?” Unfortunately, I could not get the answer.” (Code No. 17)

#### Compassion

Many students mentioned about the emotional effects of patients’ sufferings had on them.

“What caught my eye was an elderly mother of 4 sons having dreadful situation and her eyes were filled with helpless tears…. I felt very bad knowing about her condition. I wondered how it might have affected her life physically, mentally, socially and spiritually.” (Code No. 22)

“From this what I could know was how pleasant she would feel just by getting someone to listen to her, to acknowledge her and to understand her. During this conversation all we did was listening but it meant more than medications to her and at least relieved some of her emotional problems.” (Code No. 3)

“One thing that I would ever keep in my mind after this is that, if a patient visits to a hospital from a village or rural places, then it takes a lot for them to take that step and is not due to some basic illness and must be very carefully looked after.” (Code No. 53)

#### Sense of responsibility

Many reflected on the concepts of sense of responsibility and expressed how they felt towards their patients.

“I got to feel what it means to be a doctor. While seeing patient and listening to them, I have realized medical profession carries huge responsibility.” (Code No. 5)

“I feel responsible towards this patient now and I couldn’t stop blaming myself if anything happened to her. She is my patient now and I have the responsibility to cure her from every aspect.” (Code No. 19)

“I had counseled an old man, soon after I had finished he told me “you people are doing good job”. He was satisfied and walked out happily. I felt if we show respect and communicate properly with our patient; people will appreciate our effort.” (Code No. 49)

### Stage 4: active experimentation

Almost all the students were able to translate past experiences and concepts into real action. Some actions were in the form of real activities to be undertaken immediately as a student, during their stay in the PHCCs. While others were in the form of future plans when they become doctors. They also expressed their future expectations from the institution and the policymakers.

#### Immediate future plans

Based on the prior knowledge and concepts, many students planned for immediate actions to be taken during their stay at PHCCs.

“Now I’ve made up my mind, I will call her up for a follow up visit. I will do everything and anything to make her feel better and make her pain go away.” (Code No. 25)

“I should have taken a detailed history of the patient I examined. Now, I will improve on that aspect.” (Code No. 52)

“I now feel that I am able to write and I feel more responsible to be careful and not mess it around next time again.” (Code 53)

#### Suggestions to improve curriculum

Many mentioned that the common problems that they would encounter in rural settings should be incorporated in the curriculum.

“I found out that most of the patients were coming with chief complaint of fall injury. Topography of the villages is a major reason for the fall injuries. So we should be able to manage different cases related to orthopedics before going to rural districts after completing our course. It is also worthy to give training to MBBS doctor to manage different obstetrics and gynecology complications before sending them to serve the rural underserved community.” (Code No. 13)

“I saw antibiotics were indiscriminately prescribed. I thought this give rise to drug resistance organisms. I found it necessary to inculcate current status and possible situation of drug resistance in our curriculum.” (Code No. 11)

#### Plans after becoming a doctor

Many were able to internalize the experiences and transform them into active planning. They were able to reflect on what would they like to do when they become a doctor.

“In the near future, we will find ourselves in the similar scenario working in the resource poor settings. I will try my best to upgrade the quality of the lab tests. This will help the people to get the quality care in the community level.” (Code No. 18)

“Making balance between patient’s discomfort for follow up, compliance with standard treatment protocols and patient’s demands and economic status is a very difficult technique. I will have to master if I am going to work in this setting.” (Code No. 24)

“I so wish I can do something in future as a doctor to educate all my Nepalese brothers and sisters about healthy living issues, education, health, risk of teenage pregnancy and more.”(Code 51)

#### Suggestions for improving policies

Many highlighted the importance of improving the proper functioning of health facilities and expected future changes in the policies.

“The doctor was not able give full effort as per his knowledge. This is because of non-functioning of some equipment (X-ray). So in conclusion, there should be balance between human resource and equipment.” (Code No. 28)

“There is sanctioned post of medical officer but in our PHCC, there is no doctor. Not only in our PHCC, there are many PHCCs without doctor. Government should think about it and should try to find out the reason behind this.” (Code No. 26)

“We saw few times, patients have to wait quite a long time for the health workers to be in emergency room. It would be better, if one of the staffs were available continuously in the emergency. For this, co-ordination between the staffs, management committee and the community is required.” (Code No. 25)

Many students noticed that the cleanliness and sterility was not maintained in their posted health facilities. They realized that more efforts to raise awareness among the health workers were necessary.

“I think government should provide adequate materials and knowledge required to maintain sterile wound care. On one hand the patient is getting health service and on the other hand is being exposed to risk of infection.” (Code No. 14)

Some students mentioned that the data recording system was extremely poor and suggested that data management should be improved.

“Reporting of wrong and false data is being done in some settings. To minimize this, proper monitor and supervision must be done regularly.” (Code No. 25)

“I saw many patients coming for services were given new registration number and a new card although they are on follow up. If patient had brought their old cards it would have been very easy for the treating doctor. I felt it should be managed properly.” (Code No. 36)

Many students highlighted the need for better referral system and better planning of the health system.

“I felt that referral system should be more organized so that the patients do not have to roam about hospitals while in need. There should be connections among governmental and non-governmental organization regarding services like ICU, dialysis and many other services which are high resource demanding and very crucial at times.” (Code No. 24)

“Looking at the birthing center, I was surprised why on earth we are wasting so much of money. At a place where 5 deliveries are not conducted in a month, we are building delivery center with 5 rooms. It would have been better to have a multipurpose building, which could come to use instead of remaining vacant for almost all the time. Proper planning and homework is needed before budget allocation for any purpose.” (Code No. 14)

## Discussion

According to Kolb’s theory, there are two dimensions of experiential learning that take place in the brain: perceiving and processing [20]. In this study, perceiving occurred in the first stage of their experiential learning when the students had the concrete experiences both in the forms of observing the ground realities and working under resource constraint settings. These concrete experiences were then processed and transformed into knowledge in the form of reflections and subsequent planning of future actions.

In the first stage of Kolb’s experiential learning, the learners go through a concrete experience [20]. In this study, the students experienced the challenges through activities and observations. They had opportunities to observe from the patients’ perspectives and also to learn to perform various tasks in PHCC as a service provider. Previous qualitative studies on medical students’ experiential learning about rural health in other countries like Canada, Australia and South Africa have suggested that exposure to ground realities beyond tertiary care settings is an important element in learning [27–29]. Such experience can provide them an environment to understand not only the initial presence of disease and treatment, but also the importance of health promotion and social aspects of health [29, 30].

In the second stage, the students were able to review the real experiences to understand their value as suggested in Kolb’s theory [20]. The students were able to differentiate the clinical approach in primary health care and tertiary health care. They were able to reflect back on the difference between theoretical knowledge and their practical application in PHCCs. Even though these differences are known facts [28], early internalization of such differences could be important to prepare them to work in different settings in the future.

In the third stage, the learners go through a deeper reflective process, and transform the past experiences into new conceptions or knowledge [25]. In this study, the students were mainly able to understand and internalize the concept of compassion [31], by reflecting a deep awareness of other’s suffering and a strong wish to release that suffering. It has been suggested that, in order to deliver quality health care to the underserved population in rural areas, it is important to understand the values beyond the conventional clinical medicine [32]. Furthermore, the students in this study showed more realistic conceptions of the complexities of existing problems in rural areas. Such early realization can influence and better prepare those medical students with an entirely urban background to choose to work in rural areas [33, 34].

In the fourth stage, the medical students were able to use their experiences from the prior stages for planning their immediate future actions [20, 25]. They also were able to reflect upon their attitudes and the activities they would embody when becoming doctors. They also expressed future expectations and suggestions for the institutions and policymakers. These plans could lead to the next cycle of experiential learning through the lessons they learnt during this experiential cycle, to enhance their growth in subsequent years of their medical education [20].

This study has certain limitations. Firstly, the reflective writing was part of the curriculum, so the students were aware of the fact that their writing would be read by the teachers. In the South Asian context and most traditional schools, where the hierarchy in academia remains very prominent, the rigidity often deprives the students of their own growth. Although, the students in this context were encouraged to think critically and write freely, the social desirability bias cannot be completely ruled out.

Secondly, we cannot conclude that these experiences would actually lead to a new cycle of learning based on the plans they made. Further studies of the same cohort would help to provide a longitudinal perspective on whether these reflections would actually lead to a new cycle of learning based on the plans they made.

Lastly, the reflections are not entirely representative of all the medical students in Nepal or other cohorts in the same institution. More studies on experiential learning from various institutions within Nepal and other low-income countries facing human resources for health crisis are necessary to provide a better comparative perspective.

Despite these limitations, this study is the first of its kind in Nepal to explore the medical students’ reflections upon their experiential learning from PHCCs. Reflective writing of experiential learning can be an important step to address the gaps in medical education in countries like Nepal, where the perspectives of what it actually is like to learn from community settings have not yet been given adequate attention.

## Conclusion

In this study we used medical students’ written reflections to demonstrate the four stages of experiential learning in a low resource rural setting. Reflective writing in experiential learning should be encouraged as part of a medical education for future doctors that plan to practice in resource constrained settings and in low-income countries.
